# Complete resolution of non-tuberculous mycobacterial pulmonary nodule following cryobiopsy: The first case report

**DOI:** 10.1186/s13000-025-01644-z

**Published:** 2025-04-11

**Authors:** Sung Joon Han, Chaeuk Chung, Dongil Park

**Affiliations:** 1 Department of Thoracic and Cardiovascular Surgery, Chungnam National University Hospital, Daejeon, South Korea; 2 Department of Internal Medicine, College of Medicine, Chungnam National University, Daejeon, South Korea

**Keywords:** Cryobiopsy, Mycobacterial nodule, Lung cancer

## Abstract

**Background:**

Non-tuberculous mycobacterial (NTM) lung disease presents diagnostic and therapeutic challenges, often mimicking lung cancer, tuberculosis, and other bronchopulmonary disorders. Management typically involves prolonged antibiotic treatment, but alternative therapeutic approaches for localized disease remain underexplored. Cryobiopsy has emerged as an advanced bronchoscopic diagnostic technique, providing larger, higher-quality lung tissue samples compared to traditional forceps biopsy. However, its potential therapeutic effects, particularly its unintended cryoablation effect, remain an area of active investigation.

**Case presentation:**

A 46-year-old healthy woman was incidentally found to have a 1.3-cm pulmonary nodule in the left anterior basal segment during a routine health examination, with no symptoms or significant medical history. Initial CT imaging raised suspicions of T1a lung cancer. Subsequent endobronchial ultrasound transbronchial lung biopsy and transbronchial lung cryobiopsy revealed granulomatous inflammation. Tests for tuberculosis and NTM, including acid-fast bacilli (AFB) smear, mycobacterial culture, and PCR for Mycobacterium tuberculosis and NTM, were negative. Following the biopsies, chest X-rays showed an enlarged shadow at the lesion, suggesting necrosis after cryobiopsy. The patient was treated with moxifloxacin, leading to symptom improvement. A final diagnosis of NTM infection, specifically Mycobacterium avium, was confirmed from bronchoalveolar lavage fluid obtained three weeks after the tissue biopsy. Remarkably, at four months post-biopsy, a chest CT scan showed complete resolution of the nodule without additional antimicrobial therapy, suggesting a potential therapeutic effect of cryobiopsy-induced cryoablation.

**Conclusions:**

NTM nodules may undergo necrosis and resolve due to the ablation effect of cryobiopsy, suggesting cryoablation as a potential option for inoperable localized NTM disease.

## Background

Non-tuberculous mycobacterial (NTM) lung disease encompasses a complex group of pulmonary conditions caused by non-tuberculous mycobacteria. These diseases present a wide range of clinical manifestations, posing significant challenges in diagnosis, especially regarding radiological interpretation. The radiographic features of NTM lung disease can mimic those of lung cancer, tuberculosis, and other bronchopulmonary disorders, thereby complicating accurate diagnosis [1].

A recent study highlighted the diverse radiological presentations of NTM lung disease, particularly the emergence of cases presenting with a solitary pulmonary nodule [2]. These findings suggest that the radiographic appearance of NTM lung disease is not limited to typical presentations such as multifocal pneumonia or bronchiectasis; it can occasionally manifest as a solitary nodule. This is particularly important for the differential diagnosis of lung cancer [3].

The primary diagnosis of NTM lung disease is predominantly obtained by conducting microbiological examination via bronchoscopy. However, in some cases, tissue biopsies are also employed for diagnosis [4]. Conventionally, radial endobronchial ultrasound-guided forceps biopsy is commonly employed for the bronchoscopic tissue examination of peripheral lesions [5].

Cryobiopsy is increasingly recognized for its role in enhancing the accuracy of lung cancer tissue analyses. This method also improves the diagnostic yield for benign lesions, often necessitating the identification of specific diagnostic findings [6]. Identifying distinct tissue characteristics indicative of conditions such as tuberculosis, NTM lung disease, or parasitic infections presents a substantial challenge, particularly in small biopsy samples [7].

We report a case in which a lesion was diagnosed as an NTM infection through cryobiopsy, and the resolution of the lesion following the procedure suggests an unintended therapeutic effect of cryoablation.

## Case presentation

**Fig. 1:**
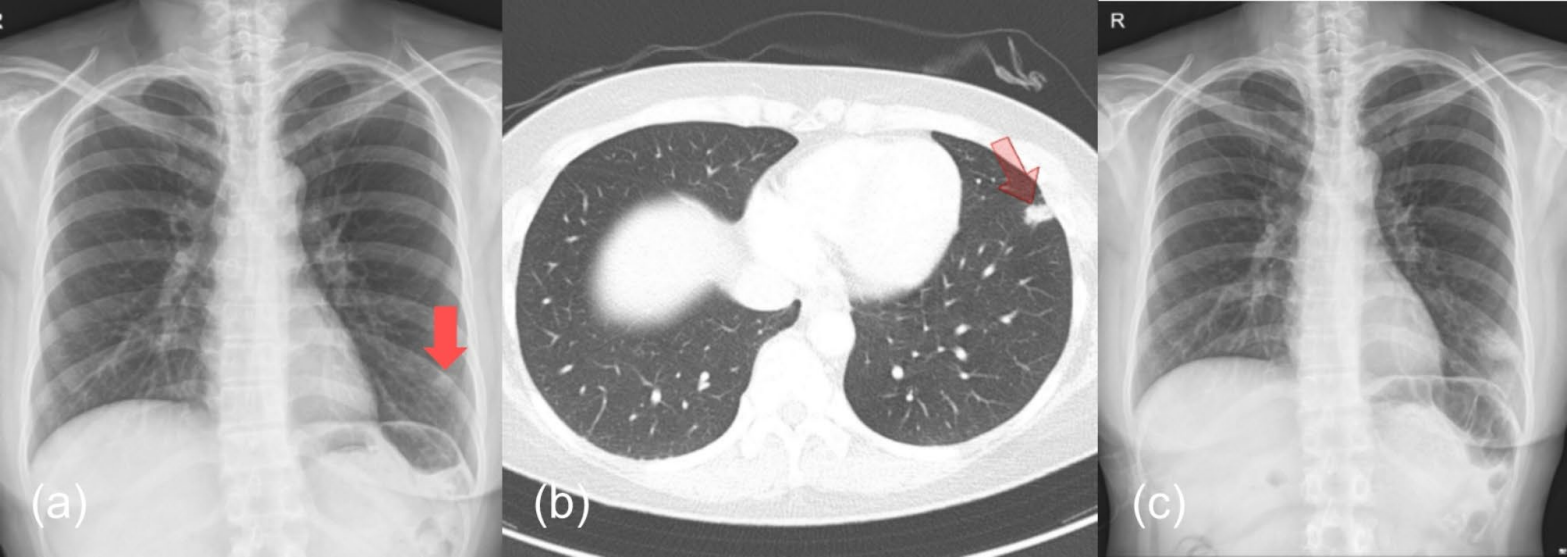
(**a**) Chest radiograph showing a solitary pulmonary nodule (solid red arrow) in the left lower lung field before cryobiopsy. (**b**) Chest CT image demonstrating a peripheral pulmonary nodule (hollow red arrow) in the left lower lobe. (**c**) Increased density in the lower zone of the left lung after cryobiopsy, as shown by chest radiograph

A 46-year-old healthy woman was incidentally found to have a 1.3-cm-sized pulmonary nodule in the left anterior basal segment during a routine health examination (Fig. 1). She reported no symptoms and had no significant medical history, including no history of tuberculosis or lung infections.

To investigate the possibility of lung cancer, the patient underwent endobronchial ultrasound transbronchial lung biopsy using a guide sheath, followed by transbronchial lung cryobiopsy employing a cryoprobe with a 1-mm diameter, performed four times through the same guide sheath. The procedure was successfully completed with only minor bleeding and no serious adverse events such as severe bleeding or pneumothorax requiring chest tube drainage.

**Fig. 2:**
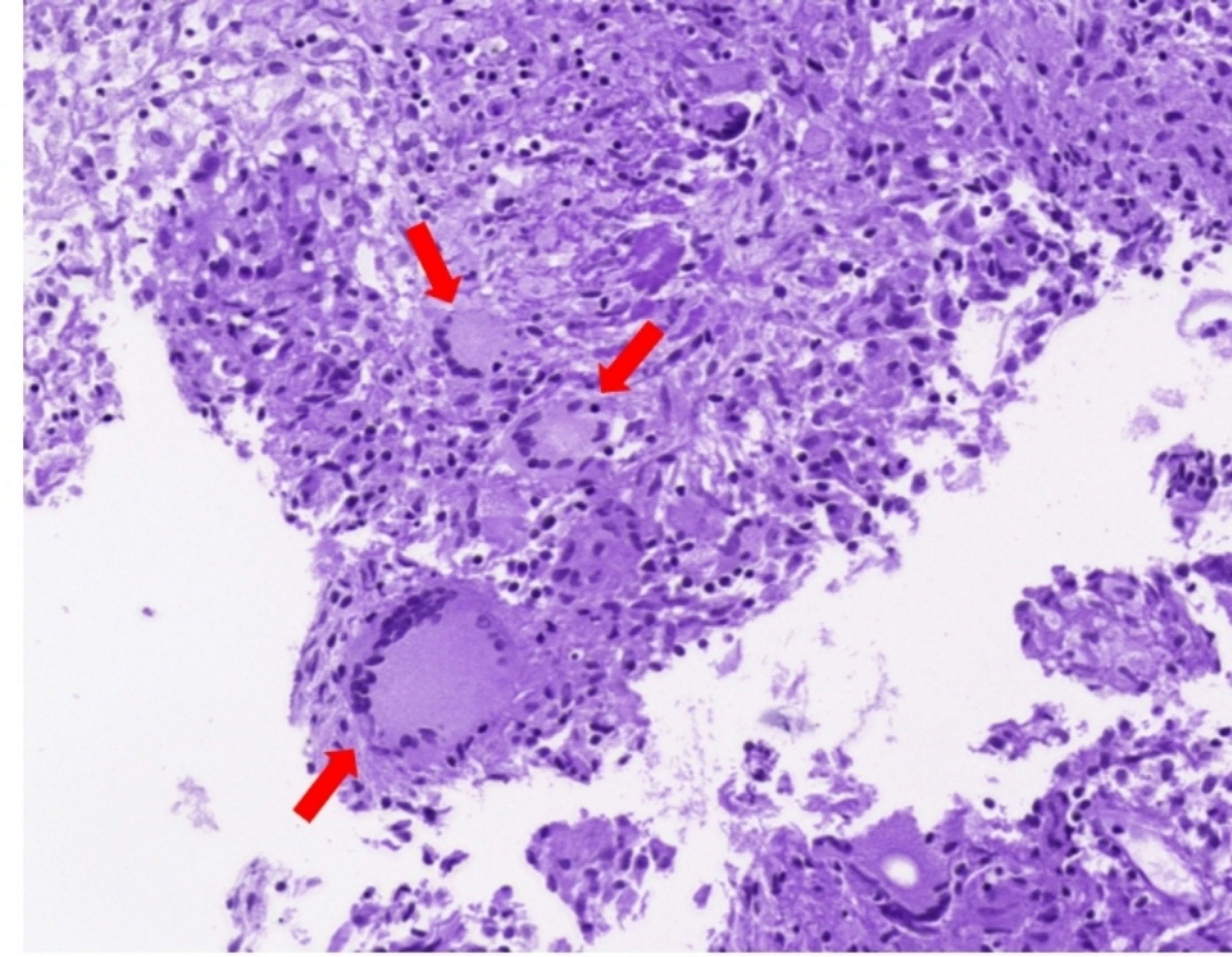
Pathologic findings of transbronchial lung biopsy (tissue biopsy obtained through cryobiopsy) revealing focal granulomas (red arrows) with infiltration of inflammatory cells (H&E stain, x40)

Biopsy revealed granulomatous inflammation in both the forceps and cryobiopsy specimens (Fig. 2). The results of the tests conducted on the tissue samples, including polymerase chain reaction (PCR) for Mycobacterium tuberculosis and NTM, yielded negative findings. One week post-biopsy, a follow-up outpatient chest radiograph revealed an enlarged shadow at the location of the original lesion, suggesting that the lesion was caused by necrosis following repeated cryobiopsies (Fig. 1). The patient reported discharge of a purulent and intermittent blood-tinged sputum and was administered a daily dose of 400 mg of moxifloxacin. Two weeks later, chest radiograph indicated a slight increase in the size of the lesion, but there was an improvement in her symptoms, with no other abnormal findings such as fever. Therefore, antibiotic therapy was continued with regular outpatient follow-ups.

Three weeks after tissue biopsy, the lesion demonstrated improvement on chest radiograph. Additionally, the growth of acid-fast bacilli was reported in the bronchoalveolar lavage (BAL) fluid obtained during examination. The final diagnosis was confirmed as NTM infection, specifically *Mycobacterium avium.*

**Fig. 3:**
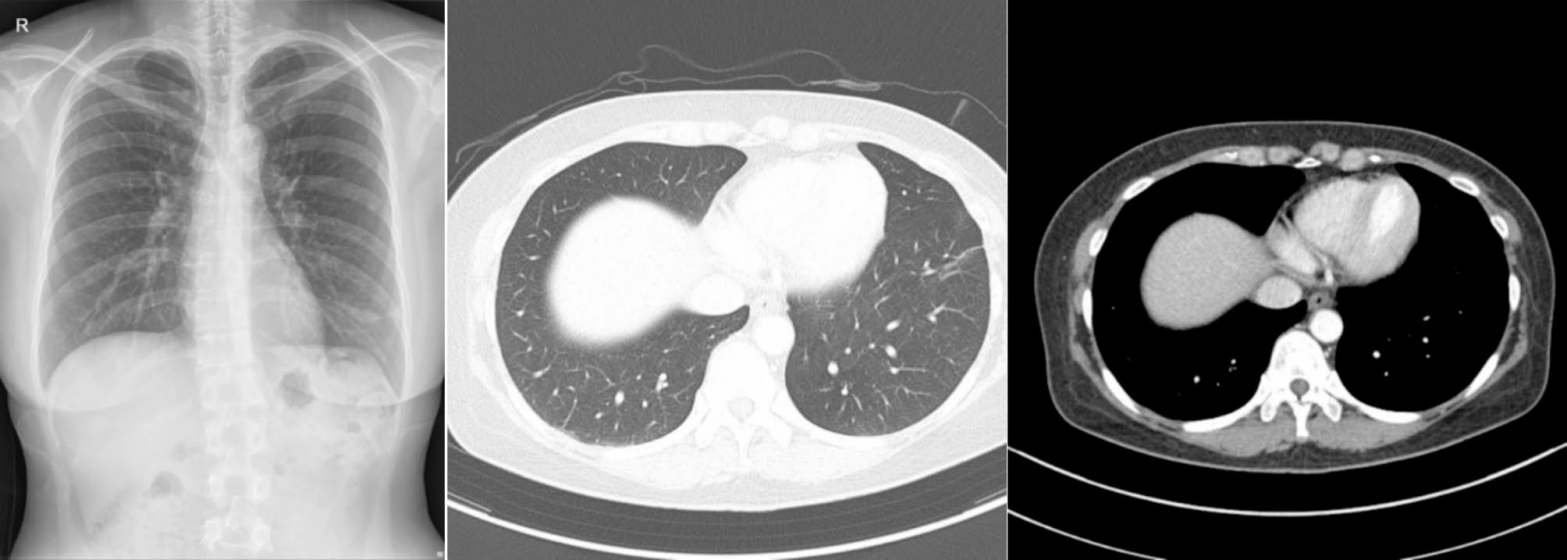
(**a**), (**b**), (**c**) Chest x-ray photograph and chest CT showing the absence of the peripheral pulmonary nodule

Four months after tissue biopsy, a follow-up chest CT scan revealed that the previously observed pulmonary nodule in the left lower lobe had disappeared (Fig. 3). Moreover, there were no signs suggestive of NTM recurrence at any other location.

## Discussion and conclusions

This case involved the incidental discovery of a pulmonary nodule, and it represents a typical example of the diagnostic challenges posed by NTM lung disease. The initial CT imaging raised suspicions of T1a lung cancer, underscoring the radiological challenges in differentiating NTM infections from malignancies. Such differentiation is crucial, considering the distinct treatment approaches and prognoses for cancer and NTM infections.

In this case, the tissue biopsy results revealed granulomatous inflammation. In lung tissue biopsies, granulomatous inflammation is frequently associated with specific infectious diseases, with tuberculosis being one of the most common causes, alongside others such as NTM, sarcoidosis, and certain fungal infections such as histoplasmosis [8, 9].

In lung cancer, granulomatous inflammation usually does not manifest directly; however, such inflammatory responses may occasionally be observed in the tissues surrounding the cancer. This could result from the impact of cancer cells on adjacent tissues, necessitating further observation and possibly re-biopsy if no specific infectious disease is diagnosed [10, 11].

In this case, the results of both tuberculosis and NTM polymerase chain reaction (PCR) conducted on the tissue were negative, and a diagnosis of *Mycobacterium avium* infection was made based on the BAL fluid specimen obtained at the time of examination. Given that the sensitivity of PCR on paraffin-embedded lung tissues is 35.3–52.9%, BAL should be considered in cases where NTM infection cannot be ruled out in lung nodules suspected of cancer [12].

Recently, local ablation therapies such as radiofrequency ablation and cryoablation have been attempted in patients with lung cancer who are unfit for surgery because of systemic conditions or advanced age [13, 14, 15, 16, 17]. Cryoablation utilizes the Joule-Thomson effect, wherein a rapid temperature drop occurs as the gas expands from high to low pressure. This results in rapid freezing of the target tissue, with ice crystals destroying the cellular structure and causing necrosis in abnormal tissues [18]. Cryobiopsy is widely used in the diagnosis of peripheral pulmonary lesions and is known to significantly increase diagnostic yield by removing normal anatomical barriers such as the bronchial mucosa, allowing for larger samples to be obtained [6, 19].

In this case, while cryoablation was not performed for therapeutic purposes, tissue samples larger than 5 mm in size in the maximum dimension were obtained four times, effectively achieving the effects of cryoablation. Unlike lung cancer, where various treatment modalities have been explored, treatment options for localized NTM pulmonary nodules have not been well studied beyond surgical intervention [14, 15, 17, 20]. However, this case suggests a potential therapeutic effect of cryobiopsy-induced cryoablation, highlighting the possibility of an alternative treatment approach for localized NTM disease that does not respond to medical therapy.

Although this case suggests a potential therapeutic effect of cryobiopsy-induced cryoablation in localized NTM pulmonary nodules, its generalizability remains uncertain. As a single-patient case study, the findings may not be widely applicable, and factors such as patient-specific immune responses, lesion characteristics, and spontaneous resolution must be considered.

In conclusion, this case demonstrates that NTM nodules may undergo necrosis and resolution owing to the ablation effect of cryobiopsy, suggesting that cryoablation might be a viable option for inoperable localized NTM disease.

## Data Availability

No datasets were generated or analysed during the current study.

## Abbreviations

BAL: Bronchoalveolar lavage
NTM: Non-tuberculous mycobacterial
PCR: Polymerase chain reaction
